# LncRNAs in the Regulation of Genes and Signaling Pathways through miRNA-Mediated and Other Mechanisms in Clear Cell Renal Cell Carcinoma

**DOI:** 10.3390/ijms222011193

**Published:** 2021-10-17

**Authors:** Eleonora A. Braga, Marina V. Fridman, Elena A. Filippova, Vitaly I. Loginov, Irina V. Pronina, Alexey M. Burdennyy, Alexander V. Karpukhin, Alexey A. Dmitriev, Sergey G. Morozov

**Affiliations:** 1 Institute of General Pathology and Pathophysiology, 125315 Moscow, Russia; p.lenyxa@yandex.ru (E.A.F.); loginov7w@gmail.com (V.I.L.); zolly_sten@mail.ru (I.V.P.); burdennyy@gmail.com (A.M.B.); sergey_moroz@list.ru (S.G.M.); 2 Vavilov Institute of General Genetics, Russian Academy of Sciences, 119991 Moscow, Russia; marina-free@mail.ru; 3 Research Centre for Medical Genetics, 115522 Moscow, Russia; karpukhin@med-gen.ru; 4 Engelhardt Institute of Molecular Biology, Russian Academy of Sciences, 119991 Moscow, Russia; alex_245@mail.ru

**Keywords:** lncRNA, clear cell renal cell carcinoma, protein targets and signaling pathways, competitive endogenous RNA model, alternative mechanisms

## Abstract

The fundamental novelty in the pathogenesis of renal cell carcinoma (RCC) was discovered as a result of the recent identification of the role of long non-coding RNAs (lncRNAs). Here, we discuss several mechanisms for the dysregulation of the expression of protein-coding genes initiated by lncRNAs in the most common and aggressive type of kidney cancer—clear cell RCC (ccRCC). A model of competitive endogenous RNA (ceRNA) is considered, in which lncRNA acts on genes through the lncRNA/miRNA/mRNA axis. For the most studied oncogenic lncRNAs, such as HOTAIR, MALAT1, and TUG1, several regulatory axes were identified in ccRCC, demonstrating a number of sites for various miRNAs. Interestingly, the LINC00973/miR-7109/Siglec-15 axis represents a novel agent that can suppress the immune response in patients with ccRCC, serving as a valuable target in addition to the PD1/PD-L1 pathway. Other mechanisms of action of lncRNAs in ccRCC, involving direct binding with proteins, mRNAs, and genes/DNA, are also considered. Our review briefly highlights methods by which various mechanisms of action of lncRNAs were verified. We pay special attention to protein targets and signaling pathways with which lncRNAs are associated in ccRCC. Thus, these new data on the different mechanisms of lncRNA functioning provide a novel basis for understanding the pathogenesis of ccRCC and the identification of new prognostic markers and targets for therapy.

## 1. Introduction

As of 2018, 400,000 reported diseases and 175,000 deaths were associated with kidney cancer worldwide [1]. Renal cell carcinoma (RCC) is diagnosed in 90% of patients with kidney cancer and has steadily increased in incidence in recent decades. High resistance to chemotherapy and a poor response to hormones, cytokines, and radiation therapy characterize it. If the disease is detected early, the recommended treatment is complete or partial nephrectomy, in which case the expected 5-year survival rate is 93% [2]. Unfortunately, about 25% of patients with RCC have metastatic tumors at the time of diagnosis and require systemic treatment. Moreover, an additional 20–50% of patients with RCC who have localized disease at baseline eventually develop metastatic RCC [3]. Targeted therapy is currently the first-line treatment for such cases. This involves the use of tyrosine kinase inhibitors (TKIs), TOR inhibitors, and monoclonal antibodies to vascular endothelial growth factor (VEGF). Recently, immune checkpoint inhibitors (ICIs) have been proposed as adjunctive treatments [3]. Unfortunately, not all patients are susceptible to both types of therapy, and over time, both targeted therapy and the use of checkpoint inhibitors can develop resistance [4,5]. This highlights the importance of the further investigation of both factors—those that influence signaling pathways involved in targeted therapy and those that regulate immune checkpoints in RCC. It is also necessary to study the mechanisms associated with other pathways that are significant in RCC, which could enable new approaches to treatment. One of the recently discovered levels of regulation is the action of long non-coding RNAs (lncRNAs), which can play the role of both oncogenes and tumor suppressors.

Numerous studies in recent years have found that lncRNAs are involved in the carcinogenesis and dysregulation of the expression of protein-coding genes in tumors through binding to chromatin modification proteins and changing their status, as well as through binding to transcription factors, RNA-binding proteins, and miRNAs [6]. These processes affect the level of synthesis of messenger RNA (mRNA) from protein-coding genes, the process of alternative splicing, the stability of mRNA, the level of translation, and protein stability [7]. Non-coding RNAs (ncRNAs) play an important role in the regulation of gene expression, ncRNA and mRNA intercommunication, and form intricate gene expression regulatory networks called competitive endogenous RNA (ceRNA) networks [8,9]. MiRNAs, a subtype of ncRNAs, silence a target gene by binding to the 3′-untranslated region (3′-UTR) of the target mRNA. LncRNAs are a type of ncRNA of more than 200 nucleotides in length. They can contain miRNA response elements (MREs) and competitively bind miRNAs that interact with other RNA transcripts containing MREs, leading to further regulation of target gene expression and complex biological processes [10,11]. LncRNAs have been implicated in RCC pathogenesis, and ceRNA-mediated mechanisms have also been reported in RCC [12]. The latest review, from 2021, examines the functions of the group of oncogenic lncRNAs and the group of suppressor lncRNAs in the pathogenesis of kidney cancer, but without detailed consideration of the various mechanisms of regulation of the protein targets and a description of the main signaling pathways affected by lncRNAs [7].

Our review discusses the most important pathways in the most common and aggressive type of kidney cancer, clear cell RCC (ccRCC), and the role of lncRNAs and target genes in signaling. Various mechanisms of dysregulation of the expression of protein-coding genes initiated by lncRNAs in ccRCC are reviewed. We describe the mechanism of competitive endogenous RNA (the ceRNA model) by which lncRNAs disrupt the regulation of the expression of genes encoding a protein, mediated by binding to regulatory miRNAs. Variants of alternative mechanisms of action of lncRNAs through direct interactions with proteins, mRNAs, and DNA sites in genes are also discussed. Since they are very diverse, the methods by which these mechanisms are established are also varied. Our review briefly highlights the methods by which various mechanisms of lncRNA action are verified. We pay special attention to the protein targets of these interactions and the signaling pathways with which they are associated in ccRCC.

## 2. Signaling Pathways and Processes Significant in ccRCC

In this chapter, we will, first of all, touch upon those signaling pathways and processes associated with the development of ccRCC, for which a significant influence of lncRNAs has been shown to date. Therefore, we do not dwell on, for example, the processes of chromatin reorganization, although mutations of the genes involved in them play an essential role in the development of this disease.

### 2.1. VHL/HIF/VEGF Pathway

**Figure 1:**
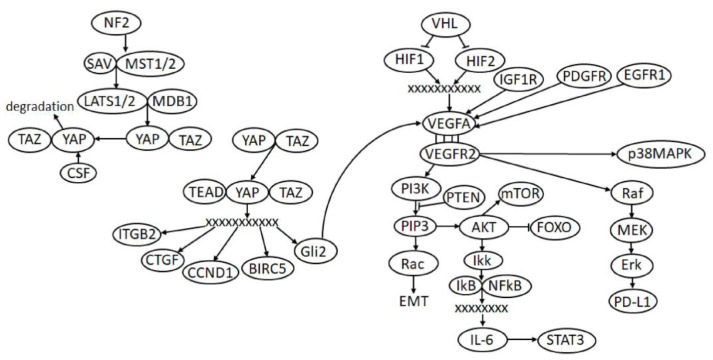
Some signaling pathways and their relationships (VHL/VEGF/HIF, PI3K/AKT, Hippo, etc.) in ccRCC. Proteins are within the ovals; straight arrows indicate activating interactions; blunt arrows indicate inhibitory interaction; the crosses indicate the interaction of transcription factors and their complexes with the regulatory regions of genes; 4 parallel small dashes show the interaction between the receptor and the ligand (VEGFA).

In ccRCC, VHL inactivation almost always occurs (due to mutations in this gene, often associated with the deletion of the corresponding region of chromosome 3 or, more rarely, its hypermethylation). VHL function is associated with ubiquitination and degradation of HIFα subunits at normal oxygen concentrations [13]. HIF2α, one of the subunits of HIFα, is located at the origin of several critical oncogenic pathways and is therefore considered an ideal target for the treatment of ccRCC. HIF2α activates genes for a variety of proteins, including angiogenic growth factors VEGFA and PDGFB, growth factor TGFα, cyclin D1, glucose transporter GLUT1, and chemokine SDF and its receptor CXCR4, which are involved in invasion and metastasis [14]. HIF2α also promotes the translation of EGFR, the TGFα receptor, as well as causing a decrease in its endocytosis [15,16]. HIF1α, which is also produced by a number of RCC lines and can be part of the HIFα complex, can also participate in the development of RCC. It also activates VEGFA and is considered one of the promising targets for therapy [17]. However, in other works, it was noted that its reactivation does not restore the ability of RCC cell lines to form a xenograft, and in a number of experiments, it generally showed oncosuppressive properties (see, for example, [18]). Despite the fact that HIF affects many signaling pathways that are essential for oncogenesis, the VEGF pathway is, of course, the most significant, which makes it an important target of therapy. Among all types of epithelial cancer, ccRCC tumors have the highest expression of VEGFA, and it is one of the most vascularized tumor types [13]. In turn, during RCC, VEGFA activates the PI3K/AKT/mTOR pathway, the MAPK/ERK pathway, as well as, directly or indirectly, many other signaling pathways [19]. Some dependencies are shown in Figure 1. In the therapy of RCC, drugs are currently widely used, the targets of which are VEGFR, PDGFR, EGFR, and other receptor tyrosine kinases [2,5].

### 2.2. PI3K/AKT/mTOR Pathway

This pathway in RCC is relatively less affected by mutations, but it is almost always deregulated and activated, and also affects a large number of pathways and processes in RCC [20,21]. Nevertheless, it should be noted that changes in the genes of this pathway (primarily *PTEN*, *MTOR*, and *PIK3CA*) are observed in 16.2% of ccRCC cases [1]. It is also important that the deregulation of this pathway correlates with the aggressiveness of RCC and a decrease in overall survival [21], whereas the nature of the *VHL* gene disorders practically does not reveal such an association [1,22]. Of the drugs used in the therapy of RCC, mTOR inhibitors act on this pathway [2].

### 2.3. Hippo Signaling

Hippo is an evolutionarily conservative signaling pathway that regulates organ size and tissue homeostasis; it affects cell proliferation, survival, differentiation, and the determination of cell fate [23]. It has also been reported that various components of Hippo can be involved in the regulation of the immune response, in some cases, ensuring the evasion of tumor cells from this response, including the participation of the immune checkpoint ligand PD-L1 [23]. This is another pathway of which the deregulation associated with overactivation is widespread in ccRCC [24]. The authors note that, in some cases, this is explained by the loss of expression of the SAV regulatory protein, but other mechanisms of disorders are also possible. One way or another, if YAP1, a key protein of this pathway, is phosphorylated, then it remains in the cytoplasm and degrades, otherwise, it passes into the nucleus and acts as a transcription factor. Knockdown of YAP1 inhibited ccRCC proliferation, migration, and anchorage-independent growth in cells. One of the genes for which YAP1 is responsible for the transcription is the oncogene *BIRC5* (survivin), a member of the IAP apoptosis inhibitor family that suppresses caspases and blocks cell death [25]. The most significant stages of Hippo are shown in Figure 1.

The review by Calses et al. notes the potential clinical relevance of the Hippo pathway in cancer [23]. The study by Rybarczyk et al. showed that deregulation of LATS1 and overexpression of YAP1 in ccRCC is associated with a poor outcome [26]. However, the impact of the Hippo pathway on tumorigenesis may be controversial. It has been shown that ferroptosis (a form of regulated cell death) is stimulated in RCC by overexpression of TAZ, a regulator of YAP1 translocation into the nucleus [27].

### 2.4. Epithelial-Mesenchymal Transition (EMT)

In EMT, epithelial cells dedifferentiate into mesenchymal cells, losing their polarity, changing the structure of the cytoskeleton and the ability to perform cell adhesion, and acquiring the ability to migrate. A key event in EMT is the loss of E-cadherin expression by the cell. Some of the EMT-associated factors directly suppress the transcription of *E-cadherin* (for example, ZEB1, ZEB2, SNAI1/Snail 1, and SNAI2/Snail 2 (also known as Slug)), and other EMT-associated factors affect its transcription indirectly (for example, Twist). Many growth factors can trigger EMT. In addition, this process is associated with various signaling pathways (including HIF/VEGF, PI3K/AKT/mTOR, Hippo, and NFκB) [28]. For example, Akt/GSK-3β/β-Catenin is one of the pathways by which the oncogenic protein Src affects EMT in RCC [29].

EMT plays an important role in the acquisition of drug resistance by the tumor [30]. In association with fibrosis, EMT predicts poorer survival in ccRCC patients [31]. EMT is a key process in metastasis. High-grade RCC is characterized by the expression of SNAIL, a key regulator of EMT. Moreover, high expression of SNAIL is a predictor of poor survival [32]. Another factor that decreases the expression of E-cadherin in RCC is c-MET [33].

### 2.5. Suppression of the Immune Response

As is well known, the process of tumor development largely depends on its microenvironment, including the number of immune cells infiltrating it, the proportion of cells with immunosuppressive functions among them, and the ability of the tumor to suppress the immune response by exposing ligands of immune checkpoint proteins on the surface [34]. Immune checkpoint molecules, such as programmed cell death protein 1 (PD-1), its ligand PD-L1, and protein 4 associated with cytotoxic T lymphocytes (CTLA-4), attenuate T cell activation in cancer and thereby induce tumor immunotolerance [13]. As discussed above, inhibitors of immune checkpoints and their ligands have been suggested as first-line therapies for metastatic RCC [3]. Unfortunately, one of the problems with this approach is related to the fact that the tumor mutational burden (TMB) in RCC is, in most cases, not so great, and not many antigens to which the stimulated immune system could react as to foreign ones are formed in the tumor. On the other hand, kidney malignancies contain a high level of tumor-infiltrating lymphocytes and therefore are considered immunologically hot tumors that are potentially capable of eliciting a strong immune response [34]. Another problem is that suppression of the immune response may be associated with other molecules for which inhibitors have not yet been developed, and not with PD-1/PD-L1 and CTLA-4 [35].

## 3. Regulatory Function of LncRNAs via the ceRNA Mechanism in ccRCC

LncRNAs are involved in the regulation of protein-coding genes through various mechanisms, including through direct interaction with mRNA or proteins. Currently, the model of competitive endogenous RNA (ceRNA) has gained wide acceptance, in which lncRNA can increase the level of protein-coding mRNA mediated by miRNA. At the same time, both mRNA of this gene and regulatory lncRNA competitively interact with miRNA. Moreover, these interactions involve MREs found in both lncRNA and mRNA, usually in the 3’-UTR of mRNA, which was first formulated in 2011 by Salmena et al. [10]. LncRNAs are primarily divided into oncogenic lncRNAs, which increase the level of expression of the pro-oncogenic protein genes regulated by them, and suppressor lncRNAs, which increase the expression of tumor suppressor genes. These effects, according to the ceRNA model, are realized through the inhibition of miRNA by lncRNA, which leads to the activation of the target gene of this miRNA. These sequential effects are schematically described by the lncRNA/miRNA/mRNA axis. To confirm the ceRNA model, it is necessary to show the mutual influences between all three components of the regulatory axis and the direct binding of miRNAs to both the mRNA of the protein gene and lncRNA. Methods used to prove these interactions and direct binding include the bioinformatic screening of target gene and lncRNAs complementary to miRNA; analysis of the expression levels of all components of the axis in clinical samples via qRT-PCR and the identification of negative correlations for the expression levels of directly interacting components and a positive correlation between the levels of mRNA and lncRNA; experiments on transfection and the analysis of mutual influences according to the gain-of-function or loss-of-function scheme; dual-luciferase testing and methods based on the principle of RNA pull-down or RNA immunoprecipitation (RIP assay). Due to the fact that interactions between different ncRNAs occur in a complex with proteins of the Argonaute family, it is desirable to confirm the RNA‒protein interaction. Validation of the direct binding of miRNA to lncRNA and miRNA to mRNA by one or another biochemical method is considered a necessary standard in modern research (see, for example, [36,37,38]).

When simulating the effect of the overexpression of a particular RNA, both in vitro and in vivo (on xenografts), as a rule, mimetics or genetically engineered constructs are used. Reducing the expression of the target gene is achieved by knocking it down with the help of the corresponding siRNAs or hairpin-forming short RNAs (shRNAs). Evidence of the biological significance of the interaction is considered to be the “cancellation” of the effect of lncRNA overexpression upon overexpression of the target miRNA and the effect of lncRNA activation upon a decrease in the expression of the miRNA to which it binds (see, for example, [38,39,40]).

These interactions are often interconnected and the understanding of this RNA crosstalk will lead to the comprehension of gene regulatory networks [8,41]. Moreover, since mature miRNAs are formed only in the cytoplasm, only lncRNAs, which are present in the cytoplasm, can participate in the mechanism mediated by miRNAs. These are discussed below.

### 3.1. The LncRNAs Acting According to the ceRNA Model

More than 40 lncRNAs have been identified in RCC, which are involved in the regulation of the mRNA level of protein-coding genes via the ceRNA mechanism. The data on the regulatory axes for these lncRNAs, as well as their influence on the pathogenesis and progression of RCC, signaling, and patient survival are given in Table 1. Only those works are presented in which the interaction of miRNA with lncRNA and with mRNA are shown using qRT-PCR, transfection, or gain-of-function/loss-of-function, and in most works, they are confirmed with direct methods as dual-luciferase and/or RIP assays as well.

As follows from the data in Table 1, more than 80% of the studied lncRNAs are oncogenic lncRNAs that activate the expression of proto-oncogenic protein-coding genes, such as *CCND1/2*. Through interactions with miRNA and mRNA along axes, oncogenic lncRNAs are involved in decreasing apoptosis, promoting migration, invasion, metastasis, and an increase in their expression correlates with a decrease in patient survival, which have been proven in vitro and in vivo (Table 1). Suppressor lncRNAs, in contrast, stimulate apoptosis, inhibit invasion and metastasis, enhancing the survival of RCC patients and sensitivity of RCC cells to chemotherapy, for example, to sorafenib (Table 1).

The majority of the studies presented in Table 1 were published in the last 5 years. A few pioneering studies were published in 2014–2016 (e.g., [44,52]). Below, we consider in greater detail several of the most recent studies in which the role of lncRNAs in the ceRNA mechanism has been validated relatively clearly in kidney cancer.

### 3.2. Oncogenic LncRNA CDKN2B-AS1 in the ceRNA Model

*CDKN2B-AS1*, also known as *ANRIL*, is located at chromosome 9p21. A significantly higher level of CDKN2B-AS1 expression was found in RCC cell lines ACHN and Caki-1, which suggests the oncogenic properties of this lncRNA [38]. Using computational algorithms, miR-141 binding sites (i.e., MREs) were identified in the CDKN2B-AS1 sequence. RCC cells were co-transfected with miR-141 and the cloned CDKN2B-AS1 wild-type binding site, which revealed a decrease in luciferase activity, indicating a possible direct binding between miR-141 and CDKN2B-AS1. CDKN2B-AS1 knockdown in ACHN and Caki-1 cell lines enhanced the apoptosis level and suppressed cell proliferation and clonogenic survival. It was also shown that CDKN2B-AS1 knockdown suppresses cell migration and invasion, and also inhibits the epithelial-mesenchymal transition (EMT), judging by the decrease in vimentin and fibronectin (mesenchymal markers) and the increase in α-E-catenin and claudin (epithelial markers) [38]. The level of miR-141 was reduced in RCC and its downregulation was associated with an advanced grade and metastasis of RCC. The decrease in the miR-141 level is at least partially caused by hypermethylation of the promoter CpG island of the *MIR141* gene (12p13.31), which has been associated with poor survival of patients [38]. Both in vitro studies and mouse xenograft models were used to show the suppressive effects of miR-141 on tumorigenicity, including migration, invasion, and EMT, as well as the pro-apoptotic role of miR-141 in RCC cells [38]. Overexpression of miR-141 through the transfection of a miR-141 mimic in ACHN and Caki1 cells reduced the CDKN2BAS1 expression level, indicating a reciprocal relationship between miR-141 and CDKN2B-AS1. Furthermore, overexpression of miR-141 or inhibition of CDKN2BAS1 reduced cyclin-D1/D2 expression at mRNA and protein levels. Importantly, the binding of miR-141 with the lncRNA CDKN2B-AS1 and with mRNAs CCND1 and CCND2 was confirmed by means of dual-luciferase and RIP assays [38]. The oncogenic effects of CDKN2B-AS1 HCP5 on RCC progression are at least partially mediated through the CDKN2B-AS1/miR-141/CCND1 and CCND2 axes (Table 1).

### 3.3. Oncogenic LncRNA HCP5 in the ceRNA Model

The HCP5 (HLA complex P5) lncRNA level was increased in RCC tissue samples and was associated with progression and the poor survival of patients [37]. Using the loss-of-function approach, it was shown that HCP5 knockdown increased the apoptotic rate, inhibited proliferation, colony formation, migration, and invasion and promoted cell cycle arrest at the G0/G1 stage [37]. As was suggested by the use of starBase, HCP5 harbored a binding site for miR-140-5p and their direct interaction was confirmed via the dual-luciferase reporter assay and the RIP-ago2 assay [37]. IGF1R (insulin-like growth factor-1 receptor) was suggested as a target of miR-140-5p in RCC via bioinformatic analysis and qRT-PCR analysis, which showed the reverse relationship between miR-140-5p and IGF1R mRNA levels among representative RCC tissue samples. Inhibition of IGF1R mRNA levels by transfected miR-140-5p mimics and the dual-luciferase reporter assay confirmed the direct interaction between them [37]. The oncogenic effects of HCP5 on proliferation and colony formation are at least partially mediated through the HCP5/miR-140-5p/IGF1R axis (Table 1). Additionally, using xenograft tumors, it was shown that downregulation of HCP5 suppressed RCC tumor growth in vivo. Importantly, the direct interaction between miR-140-5p and HCP5 or IGF1R was confirmed via the dual-luciferase reporter assay and the RIP-ago2 assay [37].

### 3.4. Oncogenic LncRNA LINC00973 as an Immune Suppressor in the ceRNA Model

As is well known, immunotherapy is the most applicable treatment in RCC, and the topic of immunity checkpoints has gained relevance in the last few years. LncRNA and miRNA, which regulate genes that suppress human immunity, are currently the most relevant, especially in the treatment of patients with RCC.

Recently, Siglec-15 has been identified as a new tumor immune suppressor, which is highly expressed in cancers [78]. Both Siglec-15 mRNA and lncRNA LINC00973 were upregulated in RCC cell lines and positively correlated with each other [35]. Moreover, Siglec-15 is better expressed on the cell surface of precisely those tumors and cell lines where LINC00973 expression is stronger. Both the 3′-UTR of Siglec-15 mRNA and LINC00973 contain the overlapping 7-6-nucleotide sites for binding miR-7109. Indeed, functional interactions of miR-7109 with LINC00973 and the 3′-UTR of Siglec-15 mRNA were suggested in gain- and loss-of-function studies and confirmed by the luciferase assay [35]. Furthermore, the direct binding between miR-7109 and LINC00973 was proven using the biotin-labeled RNA-pulldown assay. The immune-suppressive effect of Siglec-15 was confirmed via IL-2 production analysis in the Jurkat RCC cell co-culture system. This study showed that lncRNA LINC00973 is involved in immune evasion, increasing the cell surface abundance of Siglec-15 through the LINC00973/miR-7109/Siglec-15 axis (Table 1). It is important that this is an alternative mechanism of immune suppression for those tumors where the PD1/PD-L1 pathway is not expressed and drugs that suppress it do not work, but Siglec-15 can work [35]. Therefore, great hopes have been pinned on research into the Siglec-15 immunosuppressive agent and on regulators of its activity, such as miR-7109 and LINC00973.

### 3.5. Oncogenic LncRNA LINC01094 in the ceRNA Model

Chondroitin sulfate synthase 1 (CHSY1), one of glycosyltransferases, exhibits oncogenic features, promoting the progression of hepatocellular and colorectal cancers and activating the hedgehog signaling pathway and the NF-kappa-B and/or the caspase-3/7 signaling pathways [79,80]. As has been shown recently [51], LINC01094 is highly expressed in ccRCC tissues and promotes ccRCC cell growth and metastasis, activating CHSY1 by means of the FOXM1>LINC01094/miR-224-5p/CHSY1 regulatory axis (Table 1). Interactions along this axis have primarily been suggested using bioinformatics tools, such as the starBase and DIANA databases, and by loss- or gain-of-function studies using qRT-PCR and Western blotting. Direct binding of miR-224-5p with the CHSY1 mRNA has been confirmed via luciferase reporter experiments, and the direct interaction of miR-224-5p with LINC01094 was proven via luciferase reporter and RIP experiments [51]. Using an animal model, it was also shown that LINC01094 promoted tumor growth and metastasis in vivo. Moreover, LINC01094 was activated by FOXM1 at the transcriptional level. Therefore, the oncogenic properties of the lncRNA LINC01094 are at least partly implemented through the FOXM1>LINC01094/miR-224-5p/CHSY1 axis in RCC (Table 1).

### 3.6. Oncogenic LncRNA LOXL1-AS1 in the ceRNA Model

The lncRNA lysyl oxidase-like 1 antisense RNA 1 (LOXL1-AS1) is a rather novel lncRNA with oncogenic properties in several cancers, including RCC [53]. LOXL1-AS1 was upregulated in cell lines and clinical samples of RCC; knockdown of LOXL1-AS1 elevated the rate of apoptosis, suppressed the proliferation and migration of RCC cells, enhanced the E-cadherin level, and reduced the levels of N-cadherin and MMP2, markers of EMT-MET transition. To study the downstream regulatory mechanism of LOXL1-AS1 via miRNA sponge, miRNA binding sites were screened using starBase. The eight nucleotides ACCAAGAG in miR-589-5p were absolutely complementary with a site (MRE) in LOXL1-AS1. In RCC, the direct interaction of LOXL1-AS1 with the tumor-suppressive miR-589-5p was observed using the RNA pull-down assay and the luciferase reporter assay [53]. To predict the possible targets of miR-589-5p, starBase was also used. The six nucleotides CAAGAG were identified in the mRNA of CBX5 (chromobox 5) for binding with miR-589-5p, which matched six of eight nucleotides in MRE identified in the lncRNA LOXL1-AS1 for interaction with miR-589-5p. Furthermore, it was shown that the expression level of CBX5 was in proportion to the level of LOXL1-AS1 but in an inverse relationship with the miR-589-5p level in RCC clinical samples. Direct binding of miR-589-5p to CBX5 was confirmed via RNA pull-down and luciferase reporter assays. Moreover, the coexistence of LOXL1-AS1, miR-589-5p, and CBX5 in RNA-induced silencing complexes (RISCs) was shown through the RIP-Ago2 (Argonaute RISC catalytic component 2) assay. Therefore, it was proved that the lncRNA LOXL1-AS1 performs its downstream regulatory functions with the participation of the LOXL1-AS1/miR-589-5p/CBX5 signaling axis (Table 1).

### 3.7. Oncogenic LncRNA PCGEM1 in the ceRNA Model

The lncRNA PCGEM1 (prostate-specific transcript) was shown to be upregulated in RCC and involved in the progression of RCC, at least partly through the activation of fibroblast growth factor 2 (FGF2) through the PCGEM1/miR-433-3p/FGF2 axis (Table 1). The common binding sites (MREs) for miR-433-3p have been identified in PCGEM1 (7-nucleotides) and FGF2 mRNA (6-nucleotides). Direct binding of miR-433-3p with both lncRNA PCGEM1 and FGF2 mRNA has been proven using qRT-PCR, the luciferase reporter assay and the RIP-Ago2 assay [60]. PCGEM1 localization in the cytoplasm was confirmed via the FISH and subcellular fractionation assays. Gain- and loss-of-function studies were used to demonstrate mutual inhibitory effects along the PCGEM1/miR-433-3p/FGF2 axis, demonstrating a positive relationship between PCGEM1 and FGF2, and an activating effect on the proliferation and migration of PCGEM1 and FGF2, as opposed to a suppressor role of miR-433-3p in RCC [60].

### 3.8. Oncogenic LncRNA SNHG5 in the ceRNA Model

The upregulation of small nucleolar RNA host gene 5 (SNHG5) was observed in RCC cells and clinical samples. The ectopic overexpression of SNHG5 enhanced, and SNHG5 inhibition suppressed the proliferation, migration, invasion, colony formation, and EMT of RCC cells in vitro, as well as tumorigenicity and metastasis in vivo [64]. SNHG5 was preferentially distributed within the ccRCC cell cytoplasm, which is necessary for the interaction with miRNAs according to the ceRNA model. A search for regulated miRNAs and target genes revealed that both lncRNAs SNHG5 and the 3′-UTR of the transcription factor zinc finger E-box binding homeobox 1 (ZEB1) contain common sites (MREs) for binding with miR-205-5p. The relationship between SNHG5, miR-205-5p, and ZEB1 in ccRCC cells was demonstrated using the luciferase assay [64]. The RIP-Ago2 assay was also used to prove the direct binding of SNHG5 with miR-205-5p. Thus, the lncRNA SNHG5 promotes RCC progression by means of miR-205-5p downregulation and upregulation of ZEB1, according to the SNHG5/miR-205-5p/ZEB1 axis (Table 1).

### 3.9. Oncogenic LncRNA SNHG12 in the ceRNA Model

Small nucleolar RNA host gene 12 (SNHG12) expression levels were shown to be upregulated in RCC cell lines and clinical samples and were associated with lower histological differentiation, advanced stage, lymph node and distant metastases, and a shorter overall survival rate of RCC patients [65]. The knockdown of SNHG12 expression in RCC cell lines using siRNA enhanced apoptosis suppressed cell viability and invasion, confirming the oncogenic properties of this lncRNA. A negative correlation was observed between SNHG12 and miR-200c-5p expression levels, according to qRT-PCR analysis, and an effect of the transfection of si-SNHG12 and miR-200c-5p mimics suggested a reverse relationship between SNHG12 and miR-200c-5p. The application of a luciferase reporter assay confirmed an interaction between SNHG12 and miR-200c-5p [65]. Collagen type XI α1 chain (COL11A1) is associated with adhesion and extracellular matrix remodeling, which are critical processes in RCC and have significant effects on the survival of patients [81]. The common 7-nucleotide site for binding with miR-200c-5p was detected in both SNHG12 and the 3′-UTR of COL11A1 mRNA. Transfection studies using the COL11A1-WT 3′-UTR construct compared with the COL11A1-MUT 3′-UTR construct and the luciferase assay confirmed that COL11A1 is a direct target of miR-200c-5p [65]. In addition, a consistent effect on the level of apoptosis and cell viability of RCC was shown for the lncRNA SNHG12 and the mRNA of the *COL11A1* gene. According to all these data, the lncRNA SNHG12 promotes RCC progression through the SNHG12/miR-200c-5p/COL11A1 axis (Table 1).

### 3.10. Oncogenic LncRNA SNHG16 in the ceRNA Model

The SNHG16 lncRNA exhibited an increased level of expression in RCC tissues and ccRCC cells. SNHG16 has also been shown to inhibit apoptosis and promote the proliferation of ccRCC cells [66]. In addition, experiments comparing the expression levels of the SNHG16 lncRNA with predicted miRNAs and target genes, as well as transfection experiments, made it possible to determine the new axis: SNHG16/miR-1301-3p/STARD9 (StAR-related lipid transfer domain containing 9, encodes a protein belonging to the kinesin superfamily) (Table 1). Functional relationships along this axis were confirmed using the luciferase reporter assay. Moreover, direct binding of miR-1301-3p with both SNHG16 and STARD9 was proven via RNA pull-down and RNA immunoprecipitation assays [66]. Thus, SNHG16 lncRNA inhibits apoptosis and promotes RCC proliferation, at least in part, through an inhibitory interaction with miR-1301-3p, which increases STARD9 expression.

### 3.11. Oncogenic LncRNA UCA1 in the ceRNA Model

A representative set of patient samples with RCC, analyzed using qRT-PCR, showed an increased expression of UCA1 and, on the contrary, a decrease in the level of miR-182-5p [36]. Transfection of shRNA, miRNA mimics or inhibitors in 786-O and Caki-1 kidney cell lines showed reciprocal interaction between lncRNA UCA1 and miR-182-5p, as well as their opposite effect on apoptosis and RCC progression, for example, the activating effect of UCA1 and the inhibitory effect of miR-182-5p on cell proliferation, migration, and tumorigenicity [36]. In contrast, the knockdown of UCA1 inhibited RCC malignant phenotypes and Notch signaling. The potential binding sites of miR-182-5p in the lncRNA UCA1 and the 3′-UTR of Delta-like ligand 4 (DLL4) mRNA were predicted using the bioinformatics databases. Researchers also identified mismatched but overlapping 7- and 6-nucleotide sites in miR-182-5p for binding with UCA1 and the 3′-UTR of DLL4 mRNA (overlap in three nucleotides of AAC). Direct binding of miR-182-5p with UCA1, as well as with the 3′-UTR of DLL4 mRNA was demonstrated using the luciferase reporter assay [36]. Moreover, RNA immunoprecipitation (RIP) with anti-AGO2 antibodies was used to show that cells overexpressing miR-182-5p may pull down the lncRNA UCA1, thus proving the occurrence of direct interaction between UCA1 and miR-182-5p [36]. Therefore, UCA1 is involved in the inhibition of apoptosis and promotion of proliferation, migration, and RCC progression through UCA1/miR-182-5p/DLL4 axis (Table 1), and UCA1 is a potential diagnostic marker of RCC.

### 3.12. Novel Suppressive LncRNA PENG in the ceRNA Model

Only seven suppressor lncRNAs functioning in RCC according to the ceRNA model have been identified. Perhaps there are fewer of them in nature, but perhaps the interest of scientists has focused on oncogenic lncRNAs as drivers in oncogenesis. As an example, we consider the functions of a novel suppressive lncRNA, PENG, in RCC. PDZ domain containing 1 (PDZK1) belongs to the PDZ protein family and can bind to various proteins through its PDZ domain. PDZK1 is downregulated in RCC and is associated with recurrence, metastasis, and poor prognosis of patients [82]. A 6-nucleotide site was identified in miR-15b complementary to the 3′-UTR of PDZK1, and miR-15b was upregulated in RCC, and correlated with tumor size, clinical stage, and disease grade [74]. It was also shown that a high level of miR-15b promoted proliferation and shortened the overall and disease-free survival of patients with RCC. The inhibitory effect of high miR-15b on the expression level of PDZK1 was demonstrated via qRT-PCR, transfection, and gain- and loss-of-function studies in vitro and in vivo as well. The direct interaction of miR-15b with PDZK1 was confirmed via luciferase and RIP assays [74]. RNA transcribed from the ENSG00000225329.1 gene (RP11-325F22.5.1, named lncPENG) had the strongest positive correlation with the PDZK1 expression level out of five lncRNAs, which could bind to miR-15b. A number of methods were used to verify lncPENG as a novel lncRNA, for example, the size of the open reading frame or codon substitution frequency (CSF) analysis using PyhloCSF, etc. [74]. The lncPENG was downregulated in RCC and its expression decreased with the progression of cancer and was a marker of poor patient prognosis. A positive correlation was established between the lncPENG level and the PDZK1 expression level in RCC. A 7-mer seed sequence in miR-15b was complementary to the MRE on lncPENG. Using an RNA fluorescent in situ hybridization (FISH) assay, it was verified that lncPENG was located in the cytoplasm and colocalized with miR-15b. Direct binding of miR-15b with lncPENG was proven using the RIP-Ago2 assay, namely, biotin-labeled miR-15b pull-down of lncPENG [74]. Functional interactions along the lncPENG/miR-15b/PDZK1 axis were proven using the luciferase assay and a novel approach with CRISPR-Cas9-edited DICER1 [74]. It was demonstrated that lncPENG regulated the expression level of PDZK1 by competitively binding to miR-15b. Moreover, lncPENG, through the lncPENG/miR-15b/PDZK1 axis, suppressed RCC progression and improved the survival of patients with RCC (Table 1).

### 3.13. Multiple Functions of LncRNAs in the ceRNA Model

**Figure 2:**
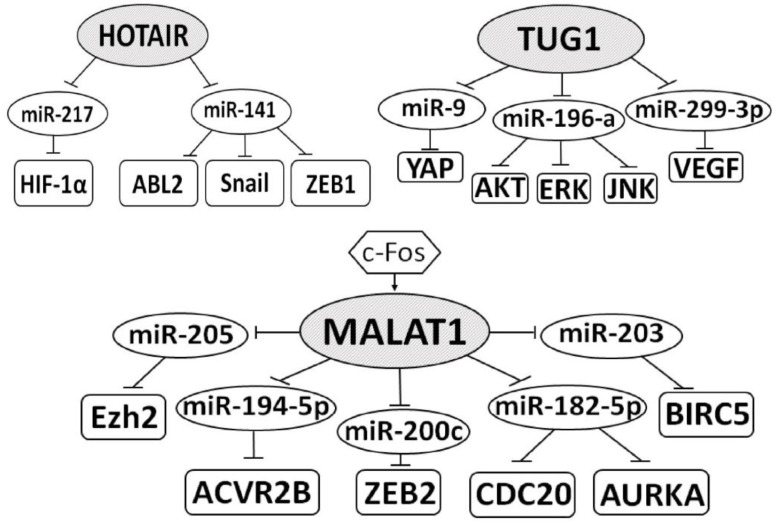
The regulatory axes of lncRNAs HOTAIR (HOX antisense intergenic RNA), MALAT1 (metastasis associated lung adenocarcinoma transcript 1), and TUG1 (taurine-upregulated gene 1), according to the following works [12,40,44,45,54,55,56,67,68,69]. lncRNAs are within the shaded ovals; miRNAs are within the unshaded ovals; proteins are within the rectangles; blunt arrows indicate inhibitory interactions; a straight arrow indicates activation of MALAT1 by c-Fos.

As shown in Table 1, for the most studied lncRNAs, such as HOTAIR, MALAT1, and TUG1, several axes have been identified, through which these oncogenic lncRNAs promote the progression of ccRCC and reduce the survival rates of patients. The axes of these three oncogenic lncRNAs are depicted graphically in Figure 2. These data also confirmed the presence of several MREs in each of these lncRNAs and multiple functions of some lncRNAs acting according to the ceRNA model.

In sum, all these data demonstrate a role for lncRNAs through nucleotide base pairing and the inhibition of specific regulatory miRNAs to protect against target mRNA suppression. Moreover, the results of these studies provide a new basis for studying the mechanism of the occurrence and development of kidney cancer.

### 3.14. Dual Features of Some LncRNAs and Their Targets or the Need to Verify Interactions for Them

It is worth mentioning that, among the regulatory axes of lncRNAs, which have been proven through a variety of methods, including luciferase and RIP assays, there are data that are difficult to interpret. Thus, in study [83], the upregulated lncRNA DLX6-AS1 promotes RCC progression via the DLX6-AS1/miR-26a/PTEN axis, although the phosphatase and tensin homolog (*PTEN*) gene is known as a classical tumor suppressor, including RCC [84]. As a result of a literature review and meta-analysis, the authors conclude that “PTEN acts as a tumor suppressor in tumorigeneses and progression in kidney cancer” [84]. Thus, the results of this work [83] require verification.

In the 2020 work [59], the authors argue that the lncRNA MIR4435-2HG promoted the progression of ccRCC through the MIR4435-2HG/miR-513a-5p/KLF6 (Krϋppel-like factor 6) axis and state that “KLF6 was dramatically increased in ccRCC”. As is known, KLF6 represents a transcription factor of the zinc finger family and plays the role of a typical suppressor of tumors of various localizations, including prostate, colorectal, etc. [85,86,87]. Oncogenic features of KLF6 in ccRCC, reported for the first time in this new study [59], may be an unexpected manifestation of the dual functions of this protein, depending on the type of cancer, but these unexpected results also seem to require verification.

## 4. Alternative Mechanisms of Regulation of the Expression of Protein-Coding Genes with the Participation of LncRNAs in ccRCC

The mechanisms of action of lncRNAs on the expression of protein-coding genes associated with oncogenesis can be varied and are not limited to the miRNA-mediated ceRNA model. LncRNAs can affect the transcription of genes and their protein products by interacting with transcription factors or by influencing chromatin reorganization. Moreover, they can bind to mRNAs, affecting their stability/expression, or bind directly to proteins, changing their stability/activity. Table 2 shows various examples of such effects in RCC. In this section, we describe not only cases in which oncogenic lncRNAs increase the expression of oncogenic proteins and tumor suppressor lncRNAs increase the expression of oncosuppressors but also cases in which oncogenic lncRNAs reduce the expression of oncosuppressor proteins and tumor suppressor lncRNAs decrease the expression of oncogenic proteins.

An extensive arsenal of methods is used to determine the interaction mechanism. As in the case of the analysis of interactions according to the ceRNA model, constructs are used for the knockdown and overexpression of the corresponding ncRNAs and proteins, as well as qRT-PCR to assess the level of expression of RNAs and Western blotting to assess the expression of proteins. As in studies of interaction through the mechanisms involved in ceRNA, clinical data are also involved and experiments are carried out on xenografts.

Some works explore different types of interactions on different cultures of RCC cells, which will be discussed below.

### 4.1. SNHG12/SP1/CDCA3 in Transcription Regulation

In study [88], it was shown that SNHG12 binds to the transcription factor SP1, stabilizing it by controlling ubiquitination, as a result of which transcription from the *CDCA3* gene promoter is enhanced. Potential SP1 binding sites on the *CDCA3* promoter were predicted using the JASPAR program. The following observations showed the binding of anti-SP1 antibodies to one of these positions. A double luciferase test was also performed, showing SP1 binding to a plasmid containing a wild-type promoter, but not to a mutation disrupting this site. RIP assays (RNA-binding protein immunoprecipitation) were used to prove the binding of SNHG12 to SP1. In addition, when using cycloheximide (CHX), a protein synthesis inhibitor in cells with SNHG12 knockdown, the amount of SP1 decreased, whereas it remained stable when SNHG12 was overexpressed. This suggests the role of this lncRNA in stabilizing the SP1 protein. Questions arose about its mechanism. The use of MG132, a proteasome inhibitor, and chloroquine, a lysosome inhibitor, suggested that binding to SNHG12 protects the protein, primarily from ubiquitination and degradation in proteasomes, since when MG132 was added, SP1 remained stable even in cells with SNHG12 knockdown. It has also been directly shown, via ubiquitination-related immunoprecipitation, that the level of SP1 ubiquitination is regulated by SNHG12 [88].

### 4.2. MAGI2-AS3/HEY1/ACY1 in Transcription Regulation

MAGI2-AS3 was shown to be bound with HEY1 and reduced HEY1 enrichment at the *ACY1* promoter region. As a result, *ACY1* expression is increased [102]. The analysis of databases (RAID v2.0, LncMAP, and RNA–Protein Interaction Prediction databases) revealed proteins that bind to MAGI2-AS3, including HEY1. A RIP-qPCR assay was performed, which revealed an enrichment of MAGI2-AS3 in the RNA–protein complex pulled down by HEY1 antibodies. HEY1 knockdown significantly reduced RCC cell survival. Analysis of databases (CistromeDB, ChIP Base, and LncMAP) also showed that *ACY1* is one of the targets of HEY1 and made it possible to predict its binding sites in the promoter region of *ACY1*. A double luciferase test confirmed this interaction. When MAGI2-AS3 was knocked down, this binding was more significant, which was accompanied by a decrease in *ACY1* expression [102].

### 4.3. HOTTIP/EZH2, LSD1/LATS2 in Chromatin Reorganization through Binding to the Gene Promoter Region

It was shown that the lncRNA HOTTIP represses *LATS2* expression by binding with the enhancer of zeste homolog 2 *(EZH2*) and lysine-specific demethylase 1 (*LSD1*) and through chromatin reorganization in the region of this gene promoter [91]. Subcellular fractionation assays showed that HOTTIP is found both in the cytoplasm and in the nucleus of cells and can influence the processes taking place there. RNA–Protein Interaction Prediction (RPISeq) software predicted the possibility of HOTTIP interaction with the EZH2 and LSD1 proteins, and then this prediction was validated using the RIP assay. Both proteins are histone methylation regulators, distributed mainly in the nucleus. Of all the potential targets of EZH2 and LSD1, HOTTIP silencing primarily increased LATS2 expression, which was confirmed both at the qRT-PCR level and using immunoblotting. EZH2 and LSD1 knockdown also increased LATS2 expression. The chromatin immunoprecipitation (ChIP) assay confirmed that the promoter region of the *LATS2* gene is occupied by EZH2 and LSD1, and suppression of HOTTIP expression reduces their ability to bind to it. In addition, histone tags H3K27me3 and H3K4me2 are found in the promoter region of the *LATS2* gene [91].

### 4.4. EGFR-AS1/EGFR in Binding to mRNA

EGFR-AS1 lncRNA has been shown to bind to EGFR mRNA, enhancing its HuR-mediated stability [95]. Thus, knockdown of EGFR-AS1 decreased the expression of EGFR mRNA and the EGFR protein itself, whereas overexpression of EGFR-AS1 increased it. Treatment with actinomycin D, a transcriptional inhibitor, enhanced the effect of EGFR-AS1 knockdown, indicating a role for EGFR-AS1 in stabilizing EGFR mRNA. The RNA fluorescent in situ hybridization (FISH) assay and immunofluorescence experiments indicated that EGFR-AS1 colocalized with EGFR mRNA. The RNA pull-down assay (selective extraction of a RNA–protein complex from a sample) demonstrated that the HuR protein is associated with both EGFR and EGFR-AS1 mRNAs, and the association of these two RNAs with HuR was also confirmed by RIP assays. As is known, HuR (synonym ELAVL1) regulates mRNA stability by binding to AU-rich elements (AREs), which are also present on the EGFR mRNA. It has been demonstrated using RIP assays that knockdown of EGFR-AS1 decreases the ability of EGFR to bind with HuR. HuR knockdown decreased EGFR expression and EGFR mRNA stability, whereas the effect of HuR overexpression was inverse. The overexpression of HuR restored the stability of EGFR mRNA, reduced by the knockdown of EGFR-AS1, and the knockdown of HuR eliminated the stimulating effect of the overexpression of EGFR-AS1 on proliferation and metastasis [95].

### 4.5. MALAT1/Livin in Binding to Protein

MALAT1 binds to the Livin protein, increasing its stability [96]. Knockdown of MALAT1 does not affect the expression of mRNA Livin but significantly reduces the expression of the protein itself. The RNA pull-down assay showed that Livin is directly related to MALAT1. CHX, a protein synthesis inhibitor, significantly reduced Livin expression, whereas MG132, a proteasome inhibitor, increased it. In cells with MALAT1 overexpression, MG132 did not affect Livin expression. MALAT1 knockdown decreased cell survival and increased apoptosis, but this effect was abolished by Livin overexpression [96].

### 4.6. Alternative Mechanisms of Action of LncRNAs

As shown in Table 2, the analysis of the regulation of protein-coding genes with the participation of suppressive lncRNAs revealed two variants of alternative mechanisms: direct binding to proteins and direct binding to mRNA as well [103,105,106]. Notably, the classification given in Table 2 is inevitably conditional, since many of the proteins that lncRNAs bind to are transcription factors or stabilize some mRNAs.

On the other hand, it can be seen that the target proteins and signaling pathways that these lncRNAs act on overlap significantly with those regulated through interactions in the ceRNA model (compare the data in Table 1 and Table 2). In addition, in both the ceRNA model and in alternative mechanisms, significantly more oncogenic lncRNAs were detected than oncosuppressive ones (see Table 1 and Table 2).

As we can see, the currently used methods make it possible to convincingly show the variety of mechanisms through which lncRNAs are involved in the regulation of the expression of genes and their products and affect the development of disease in patients with RCC.

## 5. Effect of LncRNAs on Key Pathways and Processes in ccRCC

Let us briefly characterize lncRNA targets, the effects of which have been shown in RCC, and the pathways and processes in which they are involved. It is noteworthy that most lncRNAs are oncogenic, and as a rule, they increase the expression of oncogenic proteins. Considering that the most common and well-known disorders in RCC usually involve oncosuppressive genes (and are often associated with deletions of chromosomal regions), this provides additional understanding of the mechanisms of development of the disease and the possibilities of influencing them.

### 5.1. LncRNAs in VEGF Signaling

Of the oncogenic lncRNAs, VEGF expression is increased by ROR [61] and TUG1 [68]. The oncogenic effect of LINC01234 through the effect on the HIF-2α pathway has also been reported [108]. In addition, HOTAIR increases the expression of HIF-1α, and it is significant that this effect is oncogenic [45], although the nature of the effect of HIF-1α in RCC, as already mentioned, has been debated. Even more remarkable is the recent work on the oncosuppressive antiangiogenic effect of MAGI2-AS3. Although it is not very clear how the resulting increase in the expression of the ACY1 (aminoacylase 1) protein works, the possibility of influencing the process, which is so important in ccRCC, potentially opens up prospects of interest [102].

### 5.2. LncRNAs in PI3K/AKT/mTOR Signaling

The oncogenic lncRNAs lncARSR [52], TUG1 [67], URRCC [90], FGD5-AS1 [109], LINC00982, DUXAP9 [110], DLEU1 [111], LUKAT1 [112], MALAT1 [113], and HOTTIP [114] are described as activators of the PI3K/AKT/mTOR pathway in RCC. The oncosuppressive lncRNA SARCC is known to inhibit this pathway [106]. LncTCL6 is also a tumor suppressor, which inactivates AKT- and Src-mediated EMT through its effect on Src degradation [104]. In many works, the pathways through which AKT activation proceeds are not described, and often specific protein targets have not been found either; however, a significant number of works indicate that many processes in the RCC occur through the AKT pathway or affect it. The study by Liu et al. [115] stands apart because it states that TP73-AS1 exerts a pro-oncogenic effect by inactivating the AKT pathway. The authors did not even discuss the contradictions between their data and what is already known about the RCC, so we did not include their work in Table 2. The study by Zeng et al. [83] also contains problematic results, since, in that work, DLX6-AS1 acts as an oncogene and, through the ceRNA mechanism, increases the expression of PTEN, a well-known suppressor gene. We also decided not to include it in our pivot tables. On the contrary, it was rightly noted in [107] that the effect of the oncosuppressive lnc-DILC on the PI3K/AKT pathway occurs through the stabilization of the PTEN protein, a negative regulator of this pathway.

### 5.3. LncRNAs in Hippo Signaling

**Figure 3:**
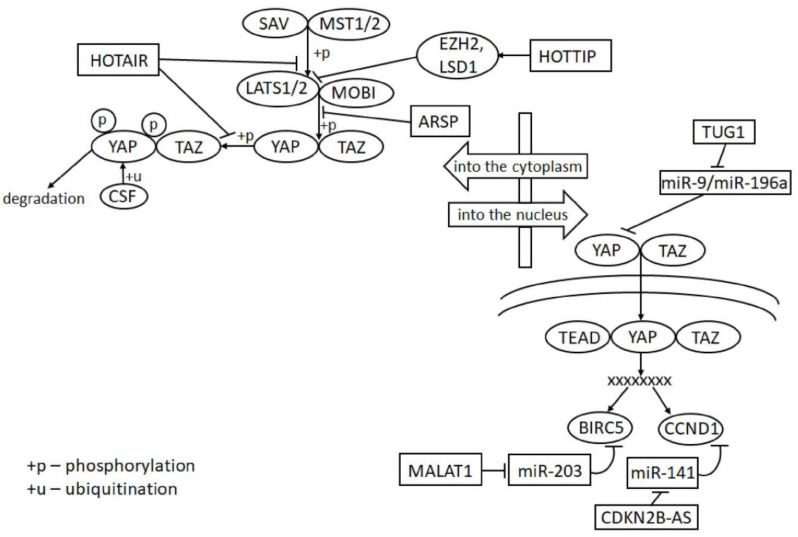
Effects of six lncRNAs (HOTAIR, HOTTIP, ARSP, TUG1, MALAT1, and CDKN2B-AS1) on different steps in the Hippo signaling pathway. lncRNAs and miRNAs are within the rectangles; proteins are within the ovals; the crosses indicate the interaction of transcription factors and their complexes with the regulatory regions of genes.

It is noteworthy that, of the lncRNAs included in Table 1 and Table 2, at least six (HOTAIR [97], HOTTIP [91], ARSP [99], TUG1 [69], MALAT1 [56], and CDKN2B-AS1 [38]) affect certain stages of the Hippo signaling pathway, as shown graphically for these lncRNAs in Figure 3. This once again emphasizes the significant role of lncRNAs in the regulation of this pathway.

### 5.4. LncRNAs in EMT Processes

Of the lncRNAs included in the same tables, that is, those for which protein targets are well known, for CDKN2B-AS1 [38], DLEU2 [43], HOTAIR [45], HOXA11-AS [47], MALAT1 [12,55], SNHG5 [64], TUG1 [68], and DUXAP9 [101], the stimulation of EMT was noted, and for NONHSAT 113026 (NOAT113026) [103] and TCL6 [104], EMT suppression was noted. Thus, this process, either directly or indirectly, is highly regulated by lncRNAs. It is interesting to consider the protein targets by which different lncRNAs affect EMT. We can observe that the mechanisms of this effect are diverse—among the target proteins, there are transcription factors that directly affect the expression of E-cadherin (ZEB1, ZEB2, SLUG), proteins of the HIF/VEGF pathway (HIF-1α, VEGF), metalloproteinases that destroy the extracellular matrix (MMP16), growth factors (IGF2BP2), as well as proteins that are essential for various signaling pathways, such as Src and NF-κB/p50. There are even more studies in which EMT markers were not detected, but there was a significant effect on the motility and invasiveness of RCC cells, which may be due to the EMT. In study [116], investigating the correlation of expression data for proteins associated with EMT and lncRNAs, a group of EMT-associated lncRNAs was isolated. Subsequently, the data on these lncRNAs were used to classify ccRCC into three groups with more or less favorable prognosis and different sensitivities to chemotherapy and immunotherapy [116].

### 5.5. LncRNAs in the Suppression of the Immune Response

There are relatively few studies on the effects of lncRNAs on the immune response in RCC patients. Critically important work [35] has been devoted to immunosuppression in ccRCC. It was observed that LINC00973 increased the expression of the cancer cell surface antigen Siglec-15, another immune suppressor. An essential point is that the expression of PD-L1 and Siglec-15 are mutually exclusive; therefore, Siglec-15 and the molecules that regulate its expression (such as the lncRNA LINC00973) are potential targets for tumor immunotherapy in cases where immunosuppression is not associated with PD-1/PD-L1.

## 6. Conclusions

To date, the important role of lncRNAs in the regulation of many genes encoding proteins, as well as a number of signaling pathways, has been experimentally shown. Comparing Figure 1 and Figure 3, we can also see that lncRNAs affect various stages of the most significant signaling pathways in ccRCC, including the VEGF signaling, PI3K/AKT and Hippo signaling pathways. At least six lncRNAs—HOTAIR, HOTTIP, ARSP, TUG1, MALAT1, and CDKN2B-AS1—affect certain stages of the Hippo signaling pathway (Figure 3).

Our analysis of the set of published data showed a significantly greater role of oncogenic lncRNAs in the regulation of genes, pathways, and processes in ccRCC than suppressor ones. Thus, the role of a number of oncogenic lncRNAs (CDKN2B-AS1, DLEU2, HOTAIR, HOXA11-AS, MALAT1, SNHG5, TUG1, and DUXAP9) in the activation of EMT and two suppressor lncRNAs (NONHSAT 113026 and TCL6) in the suppression of EMT has been established.

Many new publications have shown a variety of mechanisms for the regulatory effect of lncRNAs on ccRCC-associated genes. Using a set of methods, a model of ceRNA has been provided, in which lncRNAs activate protein-coding genes by inhibiting miRNAs, the targets of which are these genes according to the lncRNA/miRNA/mRNA scheme. For the most studied lncRNAs, such as HOTAIR, MALAT1, and TUG1, several regulatory axes have been identified in RCC, demonstrating a number of sites (MREs) for various miRNAs (Figure 2).

The discovery of a new immune suppressor, Siglec-15, and the lncRNA LINC00973, which activates Siglec-15 according to the ceRNA model, has been reported, which is very important for the immunotherapy of kidney cancer, especially in those patients in whom the role of PD-1/PD-L1 checkpoints has not been identified.

Moreover, the mechanisms of action of lncRNAs on the expression of protein-coding genes associated with oncogenesis are not limited to the miRNA-mediated ceRNA model. LncRNAs can affect the transcription of genes and their protein products, interacting with transcription factors and binding to regulatory regions of genes already at the DNA level, while at the same time, influencing chromatin reorganization. In addition, lncRNAs can directly bind to mRNAs, affecting their stability/expression, or directly bind to proteins, altering their stability/activity.

It is worth mentioning that among the works performed using a complex of methods, including methods such as the direct binding of RNA to proteins using pull-down experiments or RIP assays, there are conflicting data. For example, study [115] states that TP73-AS1 exerts a pro-oncogenic effect by inactivating the AKT pathway, and another example is provided in [83]. The DLX6-AS1 oncogene, according to the ceRNA mechanism, upregulated the expression of *PTEN*, a well-known suppressor gene. In a 2020 study [59], the authors investigated the MIR4435-2HG/miR-513a-5p/KLF6 axis, which upregulated in ccRCC KLF6, which is shown to be a typical suppressor of tumors of various localizations, including prostate tumors, colorectal tumors, etc. [85,86,87]. The oncogenic features of KLF6 in ccRCC, reported for the first time in a new study [59], may be an unexpected manifestation of the dual functions of this protein, depending on the type of cancer, but these unexpected results also need to be verified. Nevertheless, many studies have been performed showing a variety of mechanisms of regulation of genes and proteins under the action of lncRNAs in ccRCC, and their data are beyond doubt.

We have thus observed that lncRNAs in ccRCC regulate many key processes that affect the development and outcome of the disease. This makes their study fundamentally important, despite the methodological difficulties associated with the variety of mechanisms involved in their action, which means that an individual experimental scheme is often required.

The data we discuss here were obtained mainly on cell lines corresponding to the most common type of kidney cancer—ccRCC. However, there is a reason to believe that many mechanisms are universal for different RCC types that is precisely shown at the lncRNA level. Thus, the 6-lncRNA signature proposed by Zuo et al. has predictive potential for three main types of RCC—ccRCC, papillary RCC (pRCC), and chromophobe RCC (chRCC) [117]. Despite significant histological and clinical differences between RCC types, many changes occurring at the molecular genetic level affect the common pathways and processes that are influenced by lncRNAs, although different participants in these pathways and processes are changed. This allows us to hope that further search in the field of lncRNAs for potential targets for RCC therapy may be useful for its various types. Thus, changes in the genes of the PI3K/AKT/mTOR pathway (primarily *PTEN*, *TSC1*, *TSC2*, and *MTOR*) are observed in 18.9% of chRCC cases [1]. pRCC type 1 is characterized by changes associated with the increased expression of c-MET (changes in the gene copy number, activation of MET transcription, or ligand-related mechanisms) [22], which stimulates the EMT, decreasing the expression of E-cadherin. One of the most common pRCC type 2 aberrations is mutations in NF2 (Merlin-1) [1], which suppress the Hippo pathway [118].

## Figures and Tables

**Table 1:** Regulatory axes of lncRNAs acting according to the ceRNA model in ccRCC.

LncRNA/miRNA/mRNA Axis	Effect on Pathogenesis	Refs.
Oncogenic lncRNAs		
CDKN2B-AS1/miR-141/ CCND1, CCND2	migration, invasion, and clonogenicity in vitro/in vivo, RAC1/pPXN p/w, EMT, poor survival	[38]
DARS-AS1/miR-194-5p/DARS	proliferation, apoptosis inhibition	[42]
DLEU2/miR-30a-5p/ZEB2	EMT, migration, invasion, metastasis, short OS	[43]
HCP5/miR-140-5p/IGF1R	migration, invasion, metastasis, cell cycle, poor OS and PFS	[37]
HOTAIR/miR-141/ ABL2, Snail, ZEB1	proliferation, invasion	[44]
HOTAIR/miR-217/HIF-1α	migration, EMT, AXL signaling	[45]
HOTTIP/miR-615/IGF-2	decreased apoptosis, enhanced migration and invasion in vitro, metastasis, vascular invasion	[46]
HOXA11-AS/miR-146b-5p/ MMP16	invasion, EMT, advanced stage, metastasis	[47]
ITGB2-AS1/miR-328-5p/HMGA1	tumorigenesis in vitro/in vivo, poorer prognosis	[48]
KIF9-AS1/miR-497-5p/ SMAD3, ATG9A	decreased apoptosis, enhanced cell viability, resistance to sorafenib, TGF-β/autophagy p/w	[49]
LINC00511/miR-625/CCND1	metastasis, cell cycle, short OS	[50]
LINC00973/miR-7109/Siglec-15	ccRCC progression, cancer immune evasion	[35]
FOXM1→LINC01094/ miR-224-5p/CHSY1	tumor cell growth, metastasis	[51]
lncARSR/miR-34, miR-449/ AXL, c-MET	promotion of sunitinib resistance, activation of STAT3, AKT, and ERK p/w	[52]
LOXL1-AS1/miR-589-5p/CBX5	cell proliferation, migration	[53]
LUCAT1/miR-495-3p/SATB1	proliferation and invasion in vitro/in vivo, metastasis, shorter OS	[39]
MALAT1/miR-182-5p/ CDC20, AURKA	progression in vitro/in vivo, cell cycle	[40]
MALAT1/miR-194-5p/ACVR2B	cell viability, proliferation, colony formation	[54]
MALAT1/miR-200c/ZEB2	invasion and metastasis in vitro/in vivo, EMT	[55]
MALAT1/miR-203/BIRC5	invasion and migration in vitro/in vivo, cell cycle	[56]
c-Fos→MALAT1/miR-205/Ezh2	EMT, invasion, decreased apoptosis, shorter OS	[12]
MIAT/miR-29c/Loxl2	proliferation and metastasis in vitro/in vivo, poor OS and DFS	[57]
MIR155HG/miR-155-5p(-3p)/ MMP2, MMP9	invasion, migration	[58]
MIR4435-2HG/miR-513a-5p/KLF6	proliferation in vitro/in vivo, invasion, metastasis	[59]
PCGEM1/miR-433-3p/FGF2	cell proliferation, migration, apoptosis repression	[60]
ROR/miR-206/VEGF	migration, invasion, metastasis	[61]
RP11-436H11.5/miR-335-5p/ BCL-W	proliferation and invasion in vitro/in vivo, poor survival	[62]
SNHG3/miR-139-5p/TOP2A	proliferation and metastasis in vitro/in vivo, worse OS and DFS	[63]
SNHG5/miR-205-5p/ZEB1	lymph node invasion, distant metastasis, EMT	[64]
SNHG12/miR-200c-5p/COL11A1	invasion, suppression of apoptosis, poor survival	[65]
SNHG16/miR-1303-p/STARD9	proliferation, suppression of apoptosis	[66]
TUG1/miR-196a/AKT, ERK, JNK	proliferation, migration, and invasion in vitro	[67]
TUG1/miR-299-3p/VEGF	invasion, migration, EMT	[68]
TUG1/miR-9/YAP1	proliferation, migration, did not alter Hippo p/w or YAP1 protein distribution	[69]
UCA1/miR-182-5p/DLL4	migration, tumorigenicity, Notch signaling	[36]
ZFAS1/miR-10a/SKA1	migration, invasion, poor prognosis, shorter OS	[70]
**Suppressive lncRNAs**		
ADAMTS9-AS2/miR-27a-3p/ FOXO1	decreased proliferation, decreased chemoresistance	[71]
GAS5/miR-21/SOX5	sensitivity to sorafenib in vitro/in vivo	[72]
lncRNA 00312/miR-34a-5p/ASS1	apoptosis and inhibition of invasion in vitro, better OS	[73]
lncPENG/miR-15b/PDZK1	proliferation inhibition in vitro/in vivo, better survival	[74]
MEG3/miR-7/RASL11B	apoptosis, G0/G1 arrest, migration, invasion inhibition	[75]
NBAT1/miR-346/GSK-3β	migration, invasion suppression, Wnt/β-catenin p/w	[76]
XIST/miR-106b-5p/P21	G0/G1 arrest, tumor suppression in vitro/in vivo	[77]

Note: DFS—disease-free survival; EMT—epithelial-mesenchymal transition; OS—overall survival; PFS—progression-free survival; p/w—pathway signaling.

**Table 2:** Alternative mechanisms of action of lncRNAs in ccRCC.

LncRNA/Protein	Mechanisms of Action	Effect on Pathogenesis, Survival, Drug Resistance	Refs.
Oncogenic lncRNAs			
Regulation of transcription			
SNHG12/SP1/CDCA3↑	binding to SP1 and preventing the ubiquitylation-dependent proteolysis of SP1; stabilized SP1 bound to the promoter of CDCA3 and increased CDCA3 expression	tumor progression and sunitinib resistance, promoting proliferation, migration, invasion in vivo	[88]
LOC653786/FOXM1↑	enhancing the transcriptional activity of *FOXM1* gene promoter	elevating the expression of FOXM1 downstream target genes cyclin D1 and cyclin B1, promoting cell cycle progression of RCC and its growth in vitro and in vivo	[89]
Chromatin reorganization			
URRCC/EGFL7↑	enhances the expression of EGFL7 by mediating histone H3 acetylation of EGFL7 promoter	activation of P-AKT signaling, and suppressing FOXO3, promotes proliferation and metastasis	[90]
HOTTIP/EZH2, LSD1/LATS2↓	binding to the enhancer of zeste homolog 2 (EZH2) and lysine specific demethylase 1 (LSD1), and repressing LATS2 expression	cell growth and apoptosis inhibition	[91]
ATB/DNMT1↑/p53↓	binding to DNMT1 and stabilizing its expression; promoting the binding of DNMT1 to p53	promoted proliferative and migratory capacities but inhibited apoptosis	[92]
Binding to mRNA			
TASR/AXL↑	binding to the 5′-UTR of AXL mRNA and its stabilization	sunitinib resistance	[93]
AR/TANAR/TWIST1↑	binding to the 5′-UTR of TWIST1 mRNA and inhibition of its nonsense-mediated mRNA decay (NMD)	vasculogenic mimicry and metastasis	[94]
EGFR-AS1/HuR/EGFR↑	binding to EGFR mRNA and enhancing HuR-mediated mRNA stability	promoted cell proliferation and invasion in vitro and in vivo	[95]
Binding to protein			
MALAT1/Livin↑	binding to Livin and enhancing its stability	promotion of proliferation and metastasis	[96]
HOTAIR/SAV1↓	binding to the SAV1 protein inhibits its interaction with MST1/2, activation of LATS1/2, and subsequent phosphorylation of YAP1	promotion of RCC development and growth by activating the Hippo pathway through direct binding to the SAV1, as a result—promotion of YAP1 translocation to the nucleus	[97]
SRLR/NF-κB/IL-6↑	binding to NF-κB	binding to NF-κB and promoting IL-6 transcription, leading to the activation of STAT3 and the development of sorafenib tolerance	[98]
ARSR/YAP1↑/ARSR	binding of lncARSR to YAP1 impedes LATS1-induced YAP1 phosphorylation and facilitates YAP1 nuclear translocation, reciprocally, YAP1/TEAD promotes lncARSR transcription	self-renewal, tumorigenicity and metastasis, tumor-initiating cells properties	[99]
THOR/IGF2BP1↑/ IGF2↑, GLI1↑, MYC↑	THOR directly associates with insulin-like growth factor 2 mRNA-binding protein 1 (IGF2BP1) to promote mRNA stabilization of IGF2BP1-regulated genes, including *IGF2*, *GLI1*, and *MYC*	cell growth, viability, and proliferation	[100]
DUXAP9/IGF2BP2↑	methylation at N6-adenosine, binding to IGF2BP2, which increases its stability	cell proliferation and motility, EMT, activate PI3K/AKT pathway and Snail expression	[101]
**Suppressive lncRNAs**			
Regulation of transcription			
MAGI2-AS3/HEY1/ACY1↑	binding of MAGI2-AS3 with HEY1 and reducing the HEY1 enrichment at the *ACY1* promoter region, increasing *ACY1* gene transcription	overexpression of MAGI2-AS3 reduces ccRCC cell viability and migration, inhibits vessel-like tube formation of HUVECs in vitro, and represses tumor growth and angiogenesis in vivo	[102]
Binding to mRNA			
NONHSAT 113026 (NOAT113026)/ (NF-κB/p50) ↓, SLUG↓	binding to the 3′-UTR of mRNA for NF-κB/p50 and SLUG and reducing their expression	inhibits the ability of cell migration, invasion, proliferation, colony formation, EMT in vitro, tumorigenesis in vivo	[103]
TCL6/miR-155/STAU1/Src↓	recruiting STAU1 and mediation of Src mRNA decay, the interaction between miR-155 and lncTCL6 attenuates this process	repress of cell proliferation and migration/invasion, EMT and induced cell-cycle arrest and apoptosis, inhibits Src-Akt-EMT network	[104]
Binding to protein			
FILNC1/AUF1↓/ c-Myc↓	interaction of FILNC1 with AUF1, a protein that binds c-Myc mRNA, and sequestering of AUF1 from the binding of c-Myc mRNA. This leads to suppression of the c-Myc protein expression	energy stress-induced apoptosis, inhibition of Warburg effect and tumor development	[105]
SARCC/AR↓/miR-143-3p↑	binding and destabilizing AR protein, preventing AR movement from the cytoplasm to the nucleus, preventing AR from interacting with HSP90	AR could directly decrease miR-143-3p, so, de-repressing miR-143-3p expression entails the expression inhibition of AKT, MMP-13, K-RAS, and P-ERK, attenuation of cell invasion, migration, and proliferation in vitro and in vivo	[106]
lnc-DILC/WWP2/USP11/PTEN↑	repressing PTEN ubiquitination through blocking the interaction between PTEN and E3 ubiquitin ligase WWP2 and recruiting the deubiquitinase USP11 to PTEN.	inhibits cell proliferation, migration, and invasion	[107]

Note: ↓—decreased expression/activity; ↑—increased expression/activity.

## Data Availability

Not applicable.
